# Simultaneous bilateral total knee arthroplasty lowers reoperation and cost at the expense of higher complications and mortality: a meta-analysis and systematic review

**DOI:** 10.1186/s43019-025-00297-y

**Published:** 2025-10-22

**Authors:** Marc Boutros, Guy Awad, Gebrane Abou Mjahed, Elie Mansour

**Affiliations:** 1 Université Saint-Joseph de Beyrouth, Beirut, 11-5076 Lebanon; 2 Division of Hip and Knee Joint Reconstruction, Department of Orthopaedics & Rehabilitation, Yale University School of Medicine, 47 College St, New Haven, CT 06510 USA

**Keywords:** Bilateral total knee arthroplasty, Simultaneous TKA, Staged TKA

## Abstract

**Background:**

The choice between simultaneous and staged bilateral total knee arthroplasty (TKA) remains controversial due to safety and resource considerations. This meta-analysis compared outcomes between the two approaches.

**Methods:**

A total of 42 comparative studies published from 2001 to 2025 were included. A combined population of 567,915 patients was analyzed, with 225,181 undergoing simultaneous and 342,734 staged bilateral TKA. Random- or fixed-effects models were used to pool data across multiple clinical end points. Outcomes included in-hospital, first-year, and 1–2-year complications, mortality, functional outcomes, reoperations, persistent pain, and healthcare utilization metrics. Effect estimates were summarized using odds ratios (OR) for dichotomous outcomes and mean differences (MD) or standardized mean differences (SMD) for continuous outcomes, all with 95% confidence intervals (CI).

**Results:**

Simultaneous TKA was associated with significantly higher odds of transfusion (OR 3.99; 95% CI 3.10–5.13; *p* < 0.001), first-year neurological complications (OR 1.48; 95% CI .128–1.71; *p* < 0.001), and first-year mortality (OR 2.43; 95% CI 2.02–2.92; *p* < 0.001). Pulmonary complications were significantly higher between 1 and 2 years postoperatively (OR 1.41; 95% CI 1.11–1.80; *p* = 0.005). However, joint infection (in-hospital, OR 0.59; 95% CI 0.40–0.89; *p* = 0.01), first-year periprosthetic fracture (OR 0.46; 95% CI 0.38–0.57; *p* < 0.001), and overall reoperation rates (OR 0.65; 95% CI 0.61–0.69; *p* < 0.001) were significantly lower in the simultaneous group. No significant differences were observed in functional scores, persistent pain, arthrofibrosis, knee instability, or extensor mechanism failure (*p* > 0.05). Simultaneous procedures were also associated with shorter operative times (MD −66.83 min; 95% CI −91.80 to −41.86; *p* < 0.001) and lower in-hospital costs (MD −$7062.67; 95% CI −13,927.78 to −197.56; *p* = 0.04).

**Conclusions:**

Simultaneous bilateral TKA offers advantages in operative efficiency, cost reduction, and lower reoperation and fracture rates, but carries increased odds of neurological complications, transfusion, and early mortality. Careful patient selection and perioperative management are essential to balance these trade-offs when considering simultaneous procedures.

## Introduction

For patients requiring bilateral knee replacements, the choice between simultaneous (one-stage) bilateral total knee arthroplasty (TKA) and staged (two-stage) bilateral TKA remains a subject of ongoing debate. In a simultaneous bilateral TKA, both knees are replaced under one anesthesia and hospitalization, whereas a staged approach entails two unilateral TKAs performed on separate occasions, ranging from days to months apart, with each surgery conducted under separate anesthesia. Even when performed just days apart, procedures involving two distinct anesthetic events are classified as staged bilateral TKAs.

Each strategy offers theoretical advantages and drawbacks. A one-stage bilateral procedure allows the patient to undergo a single anesthesia and operative event, with one combined rehabilitation period and shorter total length of hospitalization [1, 2]. From a health systems perspective, simultaneous bilateral TKA can be cost-effective, reducing the overall hospital expenditures and resource utilization compared with two admissions [1, 3]. Authors also report benefits such as more rapid overall functional recovery and higher patient satisfaction with completing both knees at once [4]. In addition, exposure to nosocomial infection may be minimized by a single hospital stay, and some evidence even suggests a lower incidence of periprosthetic joint infection when both knees are replaced in the same setting [5].

These potential advantages must be weighed against the concerns that performing two knee replacements simultaneously increases the physiological stress and risk of perioperative complications. Extensive research has examined whether simultaneous bilateral TKA carries higher morbidity and mortality than staged surgery, but findings have been conflicting [5, 6]. On one hand, numerous large cohort analyses and meta-analyses have indicated that one-stage bilateral TKA is associated with greater immediate risks, including higher rates of cardiac events, pulmonary embolism, and early postoperative mortality, compared with staged bilateral or unilateral procedures [5, 6]. Consequently, many surgeons exercise caution in selecting candidates for one-stage bilateral TKA, often excluding older patients and those with significant comorbidities owing to the amplified risk profile [7, 8].

However, with careful patient selection and perioperative management, simultaneous bilateral TKA can be performed without a marked increase in adverse outcomes [1, 3]. Several retrospective comparisons and institutional series have found no statistically significant difference in overall complication rates between simultaneous and staged bilateral procedures in matched patient populations [1, 3]. In particular, when bilateral TKA is limited to medically optimized individuals under strict protocols, short-term morbidity and mortality outcomes have been shown to approach those of unilateral TKA [7, 9]. Some studies even report that one-stage bilateral arthroplasty is as safe as two separate surgeries [10, 11]. In addition, staged bilateral TKA carries its own disadvantages, including two exposures to anesthesia and two recovery periods.

The heterogeneity of findings in the literature, with some reports emphasizing increased risks of one-stage surgery and others demonstrating comparable safety, underscores the complexity of this issue. Differences in study design, patient selection, perioperative care enhancements (such as modern fast-track protocols), and outcome definitions have all contributed to the ongoing uncertainty. At present, there is no clear consensus on the optimal approach for bilateral knee replacements, and surgical decision-making often must be individualized, balancing the potential benefits of a one-stage TKA against the potential risks.

The present study sought to provide clarity on this unresolved question by comparing outcomes between simultaneous and staged bilateral TKA. By pooling data from diverse studies, this analysis aims to deliver a comprehensive and objective assessment of perioperative complications, mortality, and recovery period for one-stage versus two-stage bilateral knee arthroplasty.

## Methods

### PROPSERO registration

This systematic review and meta-analysis was conducted in accordance with Preferred Reporting Items for Systematic Reviews and Meta-Analyses (PRISMA) guidelines and was prospectively registered in the PROSPERO database (ID: CRD420251130476).

### Search strategy

A comprehensive literature search was conducted using four electronic databases—PubMed, Scopus, Cochrane Library, and Google Scholar—from inception through 4 June 2025. The search combined the following keywords and Medical Subject Headings (MeSH) terms using Boolean operators (“AND,” “OR”): "Arthroplasty,” “Replacement,” “Total Knee Arthroplasty,” “Knee Arthroplasty,” “Total Knee Replacement,” “Knee Replacement,” “TKA,” “TKR,” “Bilateral,” “Both Knees,” “Simultaneous,” “Concurrent,” “One-Stage,” “Single-Stage,” “Same-Day,” “Immediate,” “Staged,” “Sequential,” “Delayed,” “Two-Stage,” “Interval,” “Split-Stage,” “Concurrent,” “Split.”

### Eligibility criteria and study selection

Studies eligible for inclusion comprised randomized controlled trials (RCTs) and prospective or retrospective comparative observational studies that directly contrasted outcomes of simultaneous (one-stage) versus staged (two-stage) bilateral total knee arthroplasty in adult (> 18 years) human patients. Excluded were all noncomparative designs, case reports, narrative reviews, systematic reviews, meta-analyses, conference abstracts, editorials, and purely economic or biomechanical investigations; studies of unicompartmental or revision knee arthroplasty; studies not published in the English literature; nontotal knee procedures; and nonhuman or cadaveric models.

### Data extraction

Two independent reviewers (G.A. and G.A.M.), both coauthors of this study, extracted data from each included article using a standardized spreadsheet. Collected information included study characteristics (author, year of publication, study design, total sample size, number of patients in each group, time between staged procedures, and follow-up duration). Patient demographics and baseline characteristics were also extracted, including mean age, sex ratio, body mass index (BMI), American Society of Anesthesiologists (ASA) score, and comorbidity burden measured by validated indices (Charlson or Elixhauser).

The primary outcomes of interest encompassed both medical and surgical complications, including rates of deep vein thrombosis (DVT), cardiac events, pulmonary embolism, stroke, and mortality. Surgical complications assessed included transfusion requirements, arthrofibrosis, knee instability, extensor mechanism failure, periprosthetic fracture, and periprosthetic joint infection. Additional outcomes included persistent pain at 30 days, postoperative functional scores, and the overall reoperation rate.

### Risk of bias assessment

Methodological quality was evaluated using the Risk of Bias in Non-randomized Studies of Interventions (ROBINS-I) tool for non-RCTs and the Revised Cochrane Risk-of-Bias tool (ROB2) for the RCT [12, 13]. The ROBINS-I tool examines seven domains—confounding, participant selection, classification of interventions, deviations from intended interventions, missing data, outcome measurement, and selection of reported results—and assigns a risk level (low, moderate, serious, or critical) for each domain. The ROB2 tool evaluates five domains: bias arising from the randomization process, bias due to deviations from intended interventions, bias due to missing outcome data, bias in measurement of the outcome, and bias in selection of the reported result. Each domain is rated as low risk, some concerns, or high risk of bias.

The overall risk of bias for each study was determined by the highest risk level identified in any domain. Two investigators independently conducted these assessments, resolving disagreements through discussion or by consulting a third reviewer if necessary.

### Statistical analysis

All statistical analyses were performed using Review Manager 5.4 (Cochrane Collaboration, 2020). Continuous outcomes were analyzed using either mean differences (MD) or standardized mean differences (SMD), both reported with 95% confidence intervals (CIs), depending on the consistency of the measurements. Dichotomous outcomes were evaluated using odds ratio (OR) with 95% CIs. Heterogeneity was assessed with the *Q* test and the *I*^2^ statistic; a *p* value < 0.10 or an *I*^2^ value > 50% was considered indicative of significant heterogeneity. A random-effects model was applied in cases of substantial heterogeneity, whereas a fixed-effect model was used otherwise. Statistical significance was defined as *p* = 0.05.

## Results

### Study selection

**Fig. 1:**
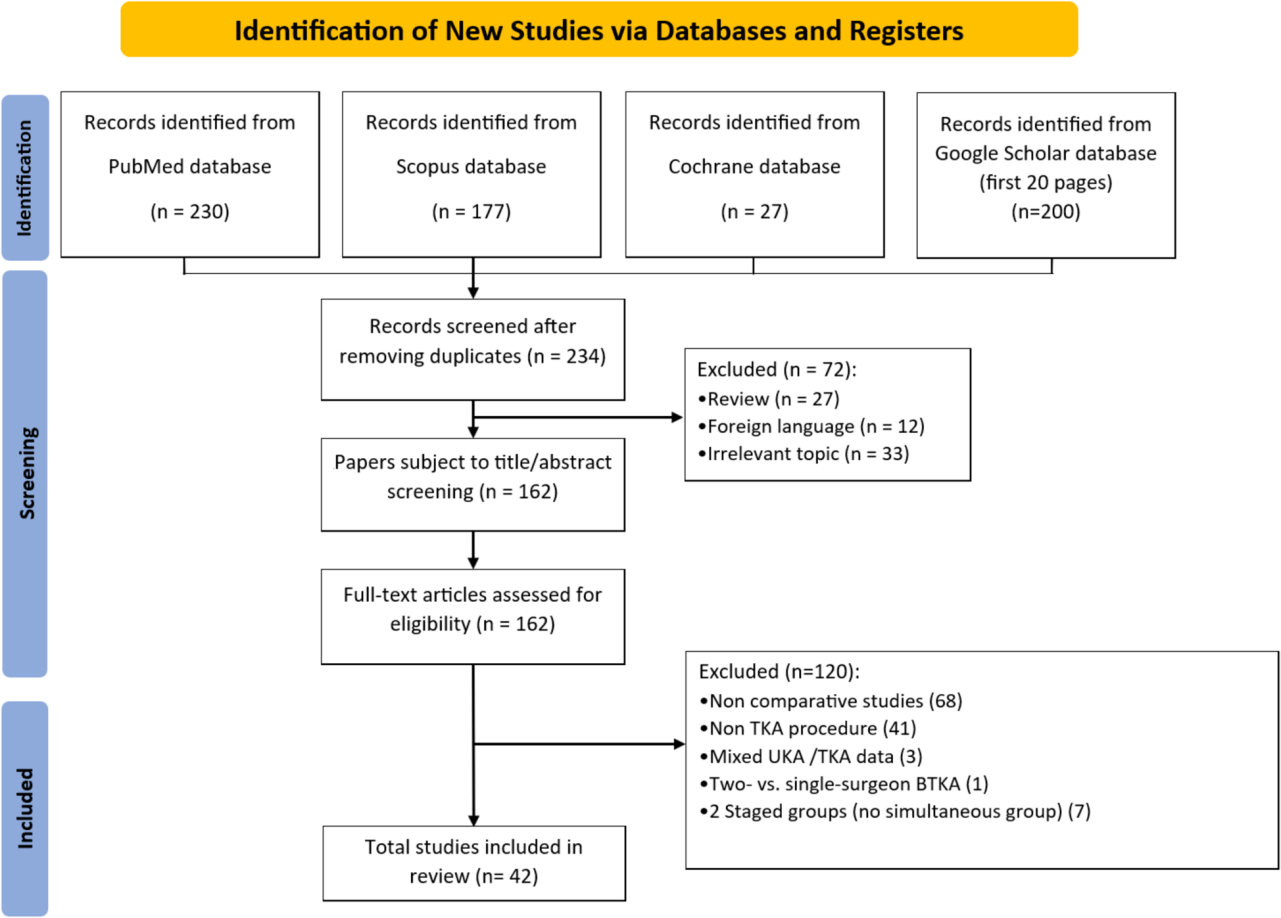
Preferred Reporting Items for Systematic Reviews and Meta-Analyses (PRISMA) flow diagram for study identification and selection

Study selection is summarized in the PRISMA flow diagram (Fig. 1). Our database searches retrieved 634 records (PubMed 230; Scopus 177; Cochrane Library 27; Google Scholar, first 20 pages, 200). After removing 400 duplicates, 234 unique records remained for title and abstract screening, of which 72 were excluded (27 reviews, 12 non-English, 33 off-topic), leaving 162 articles for full-text review. Full-text assessment led to the exclusion of 120 studies—68 noncomparative designs, 41 non-TKA procedures, 3 mixed UKA/TKA cohorts, 1 two- versus single-surgeon BTKA study, and 7 staged-only groups—resulting in 42 studies left for the final analysis.

### Characteristics of included studies

The 42 included studies (published between 2001 and 2025) comprised 1 randomized controlled trial, 2 prospective cohort studies, and 39 retrospective comparative cohorts (Table 1). All trials compared one-stage (simultaneous) versus two-stage (staged) bilateral TKA. In total, 567,915 patients were analyzed, with 225,181 undergoing simultaneous bilateral TKA and 342,734 undergoing staged bilateral TKA. Sample sizes per arm ranged from 26 to 89,568 in the simultaneous groups and from 26 to 121,115 in the staged groups, with four studies enrolling more than 10,000 patients per group. With respect to tranexamic acid (TXA) use, 9 studies reported applying a standardized TXA protocol, 9 studies explicitly stated that TXA was not used, and 24 studies did not report on TXA administration. In the studies using standardized protocols, TXA was typically administered to both simultaneous and staged groups, most often via intravenous dosing, thereby ensuring comparable exposure across treatment arms.

**Table 1:** Characteristics of included trials: design, patient demographics, interventions, and outcomes

Author	Study design	Study population (*n*)	Number of patients in simultaneous group	Number of patients in staged group	Time between staged surgeries	Follow-up duration (in months) Mean ± SD or median [IQ1–IQ3]		Measured outcomes	Use of tranexamic acid
						Simultaneous	Staged		
Singh et al. [14]	Retrospective study	210,682	*N* = 89,568	*N* = 121,115	N/A	1		Mortality; pulmonary complication; cardiac complication; transfusion; UTI; DVT; Neurological complication; osteomyelitis; sepsis; wound disruption; postop infection; dislocation; periprosthetic fracture; postop shock; 30-day readmission; 30-day reoperation	N/A
Accatino et al. [15]	Retrospective study	109	*N* = 43	*N* = 66	N/A	59.2 [12–94]	52.8 [12–78]	Not used	N/A
Ashkenazi et al. [16]	Retrospective study	410	*N* = 205	*N* = 205	N/A	63.6 (12–124.8)		Inpatient cost; length of stay; mean operative time; 30 and 90-day readmission (superficial site infection, periprosthetic joint infection); 90-day reoperation; 90-day venous thromboembolism	N/A
Chang et al. [17]	Retrospective study	2344	*N* = 1558	*N* = 786	N/A	Period from 2010 to 2020 (120 months)		30 and 90-day readmission; 30 and 90-day UTI; 30 and 90-day pulmonary complication; 30 and 90-day GI complications; 30 and 90-day cardiac complications; 30 and 90-day deep vein thrombosis; 30 and 90-day neurological complication; 30 and 90-day infection; 30 and 90-day periprosthetic fracture; 30 and 90-day instability; 30 and 90-day extensor rupture	N/A
Franceschetti et al. [18]	Retrospective study	173	*N* = 65	*N* = 108	3–12 months	12		Operative time; infection; hematoma; fracture; DVT; pulmonary embolism; pulmonary complications; cardiac complications; urologic complications; Renal failure; neurologic complications; anemia; transfusion; mortality; length of stay; 30-day readmission; forgotten joint score	All patients received an intra-articular injection of a 100-ml solution (0.9% NaCl solution, combined with 20 ml 0.5% levo-bupivacaine and 3 g of tranexamic acid) directly following capsule closure. Subsequently, 1 g of tranexamic acid was administered intravenously at 8-h intervals for the first 24 h
Kim et al. [19]	Prospective study	11,656	*N* = 7155	*N* = 4501	N/A	186	193.2	Used equally in both groups	Used equally in both groups
Matsumura et al. [20]	Retrospective study	188	*N* = 94	*N* = 94	Mean = 5 months	12		Bleeding; length of stay; operative time; transfusion; thrombosis; prosthetic infection; cardiovascular complication; renal complication; respiratory complication; GI complication; neurological complication; delayed wound healing; in-hospital and 1-year KSS and function score	Not used
Serino et al. [21]	Retrospective study	51,650	*N* = 7454	*N* = 44,196	3–12 months	Period from 2015 to 2020 (60 months)		Mortality; pulmonary complication; superficial infection; postop joint infection; renal complication; UTI; cardiac complication; wound dehiscence; hematoma; venous thromboembolism; transfusion; readmission; reoperation; ICU visit	N/A
Sustich et al. [22]	Retrospective study	73	*N* = 23	*N* = 50	N/A	6		Initial, 30-day, 90-day, 6-month MME (morphine milligram equivalents); initial, 30-day, 90-day, 6-month request for opioid refill	N/A
Tsui et al. [23]	Retrospective study	2372	*N* = 772	*N* = 1600	N/A	Period from 2001 to 2022 (252 months)		Length of stay; 30-day readmission; ICU admission; in-hospital mortality; thrombotic events; periprosthetic infection; cost	N/A
Yalin et al. [24]	Retrospective study	162	*N* = 48	*N* = 114	N/A	18.5 ± 3.2		Length of stay; postop hematoma; postop superficial infection; postop pulmonary complication; transfusion; 6- and 12-month extensor function (chair-rise test)	Tranexamic acid was intravenously infused gradually for 30 min before the surgical operation. The dose of the application was determined to be 15 mg/kg of TXA. An additional dose of TXA was administered 15 min before releasing the tourniquet
Abdalla et al. [25]	Randomized prospective study	53	*N* = 27	*N* = 26	Mean = 6.3 months	16.5 (12–20)		Urinary complication; cardiac complications; respiratory complications; neurological complications; superficial wound; weakness; postop bleeding; functional outcome (Knee Society Score)	Not used
Çelen et al. [26]	Retrospective study	231	*N* = 168	*N* = 63	Mean = 6.5 months	39.0 ± 14.7		Mean cumulative operative time; blood transfusion; 1-year range of motion; 1-year Knee Society Score; prosthesis infection; periprosthetic fraction; superficial infection; arthrofibrosis; 30-day, 90-day and 1-year mortality; pulmonary complication; cardiac complication; neurological complication	Intravenous 1 g tranexamic acid was used just before incision to reduce blood loss
Chou et al. [3]	Retrospective study	2016	*N* = 1565	*N* = 451	N/A	Period between 2011 and 2016 (60 months)		30- and 90-day readmission; 1-year readmission; transfusion; thromboembolic complication; periprosthetic fracture; surgical site complication; neurological complication; periprosthetic joint infection; periprosthetic joint infections; instability; extensor failure	Not used
Erossy et al. [27]	Retrospective study	38,764	*N* = 19,382	*N* = 19,382	N/A	Period between 2016 and 2017 (12 months)		Length of stay; cost; transfusion; periprosthetic fracture; postoperative shock; hematoma/seroma; wound dehiscence; infection; post hemorrhage anemia; pulmonary embolism; DVT; central nervous system complications; respiratory complications; cardiac complication; GI complication; genitourinary complication	N/A
Eke et al. [28]	Retrospective study	276	*N* = 225	*N* = 51	N/A	Period between 2018 and 2019 (12 months)	Transfusion; 1-month mortality; Length of stay; ICU admission	The use of tranexamic acid was relatively more common in cases that underwent simultaneous bilateral TKA than in those with staged bilateral TKA during the given study period	
Abdelaal et al. [29]	Retrospective study	2457	*N* = 2728	*N* = 729	Mean = 207.6 days	N/A		In-hospital fatalities; 90-day readmission; 1-year reoperation; 2-year revision; cardiac complications; respiratory complications; neurological complications; urinary complication; digestive complication; infection	Inconsistent use of tranexamic acid throughout study period but relatively equivalent in all groups
Mardani-Kivi et al. [30]	Retrospective study	517	*N* = 272	*N* = 245	1–12 months	24		1-month, 3-month, and 2-year function (Knee Society Score, WOMAC); patient satisfaction (VAS); genu varum; transfusion; hematoma; DVT; mortality; revision; pulmonary embolism; cerebral emboli	Intravenous injection of the tranexamic acid were used to reduce intraoperative hemorrhage
Sarazeem et al. [31]	Retrospective study	100	*N* = 51	*N* = 49	N/A	24 (12–36)		Function (Oxford knee score, WOMAC score); quality of life (KOOS score, SF-36 survey); range of motion; ICU admission; neurological complication; pulmonary complication; mortality; thromboembolic disease	Use of tranexamic acid but without further details
Gill et al. [32]	Retrospective study	168	*N* = 122	*N* = 46	Mean = 238 days	12		Length of stay; operative time; transfusion; cardiac complication; neurological complication; renal failure; infection (superficial and deep); readmission; reoperation; mortality	Not used
Richardson et al. [33]	Retrospective study	4332	*N* = 1637	*N* = 2695	6–12 months	Period between 2007 and 2015 (96 months)		30-day DVT; 30-day transfusion; 7-day cardiac complication; 7-day pulmonary complication; 7-day sepsis; 30-day bleeding; 30-day pulmonary embolism; 90-day mechanical complication; 90-day infection; 90-day readmission; 90-day manipulation under anesthesia	N/A
Tsay et al. [34]	Retrospective study	72,720	*N* = 27,301	*N* = 45,419	Mean = 181 days	12		30-day, 90-day and 1-year mortality; 30-day cardiac complication; 30-day respiratory complications; 30-day GI complication; 30-day urinary complication; 30-day hematoma; 60-day DVT; 60-day pulmonary embolism; 1-year infection (superficial and deep)	N/A
Chua et al. [35]	Retrospective study	36,087	*N* = 23,136	*N* = 12,951	Mean = 14.7 weeks	Period from 1999 to 2015 (192 months)		30-day mortality; revision; loosening/lysis; infection; patellofemoral pain; instability	N/A
Koh et al. [36]	Retrospective study	1085	*N* = 820	*N* = 265	N/A	Period from 2008 to 2014 (72 months)		Operative time; transfusion; acute kidney injury; major cardiovascular and cerebral effect; admission to ICU; 1-year mortality	N/A
Bohm et al. [37]	Retrospective study	31,602	*N* = 6349	*N* = 25,253	N/A	Period from 2006 to 2013 (84 months)		Blood transfusion; 1-, 2-, and 3-year revision; in-hospital mortality; in-hospital and 90-day complications (cardiac, pulmonary, infection)	N/A
Sheth et al. [38]	Retrospective study	22,236	*N* = 7866	*N* = 14,370	N/A	Period from 2001 to 2012 (132 months)		Revision; deep infection; cardiac complications; mortality; neurological complication; venous thromboembolism	N/A
Courtney et al. [39]	Retrospective study	234	*N* = 103	*N* = 131	N/A	17		Mortality; 90-day readmission; reoperation; blood transfusion; 1-year neurologic complication; 1-year cardiovascular complication; 1-year thromboembolism; 1-year bleeding; 1-year pulmonary complication; 1-year renal complication; 1-year infection; 1-year arthrofibrosis; 1-year extensor-mechanism complication; 1-year instability	N/A
Lindberg-Larsen et al. [40]	Retrospective study	449	*N* = 157	*N* = 292	7–18 months	Period between 2010 and 2011 (12 months)		Length of stay; 90-day mortality; 30-day readmission (infection, wound complication, DVT, cardiac complication, GI complication, UTI, pain)	N/A
Niki et al. [41]	Retrospective study	120	Simultaneous *N* = 60	Staged *N* = 60	Mean = 8.2 months	38 (24–51)		Operative time; blood loss; DVT; pulmonary embolism; cardiac complication; fracture; heterotopic ossification; neurological complication; arthrofibrosis	Intra-articular injection of tranexamic acid using the drain clamping method was employed
Bolognesi et al. [42]	Retrospective study	8307	Simultaneous *N* = 4519	Staged *N* = 3788	N/A	Period from 2000 to 2009 (108 months)		90-day, 180-day, and 1-year mortality; 90-day, 180-day, and 1-year cardiac complication; 90-day, 180-day, and 1-year infection; 90-day, 180-day, and 1-year pulmonary complication; 90-day, 180-day, and 1-year thromboembolic complication; 1- and 5-year revision; 1- and 5-year removal	N/A
Poultsides et al. [43]	Retrospective study	3976	*N* = 2825	*N* = 1151	Mean 209.6 days	Period between 2000 and 2009 (108 months)		Infection (deep and superficial, in-hospital and late); transfusion	N/A
Bini et al. [44]	Retrospective study	3336	*N* = 1220	*N* = 2116	N/A	12 months		90-day mortality; 1-year deep venous thromboembolism; 1-year pulmonary embolism; 1-year revision; 1-year infection (deep and superficial)	N/A
Yoon et al. [45]	Retrospective study	238	*N* = 119	*N* = 119	Mean = 12 months	Period from 2003 to 2006 (36 months)		Postop shock; pulmonary complication; neurological complication; renal complication; ICU admission; thromboembolic disease; mortality; superficial infection; prolonged discharge; deep infection; bleeding	Not used
Stefansdottir et al. [46]	Retrospective study	7699	*N* = 1139	*N* = 6560	>1 year	1		30-day mortality	N/A
Forster et al. [47]	Retrospective study	66	*N* = 28	*N* = 38	Mean = 29 months	57.6 (12–80.4)	46.8 (12–86.4)	Not used	Not used
Hutchinson et al. [48]	Prospective study	563	*N* = 438	*N* = 125	Mean = 34 months	76	90	Unknown	N/A
Walmsley et al. [49]	Retrospective study	2622	*N* = 826	*N* = 1796	< 1 year	Period from 1989 to 1999 (120 months)		90-day mortality	N/A
Silva et al. [50]	Retrospective study	91	*N* = 26	*N* = 65	Mean 70.5 weeks	Period from 1997 to 2001 (48 months)		Length of stay; transfusion; need for rehabilitation; cost; mortality; reoperation; cardiac complication; neurological complication; pulmonary embolism	Not used
Stubbs et al. [51]	Retrospective study	99	*N* = 61	*N* = 76	N/A	46.3	33	Not used	Not used
Ritter et al. [52]	Retrospective study	2102	*N* = 2050	*N* = 152	1.4 ± 0.8 years	192		Knee society score; deep infection; superficial infection; GI complication; thromboembolic complication; urinary complication; neurological complication	Not used
Mangaleshkar et al. [53]	Retrospective study	88	*N* = 54	*N* = 34	3–6 months	Period from 1996 to 1999 (36 months)		30-day mortality	N/A
Ritter et al. [54]	Retrospective study	44,323	*N* = 12,922	*N* = 31,401	6–12 months	Period from 1985 to 1990 (60 months)		30-day, 3-month, 6-month, 1-year and 2-year mortality; surgical complication (wound dehiscence, postop bleeding, prosthetic mechanical complication, postop wound infection); vascular complication; nosocomial infection; wound infection; cost	N/A

*N/A* Not available; UTI: Urinary tract infection; DVT: Deep vein thrombosis; GI: Gastrointestinal; MME: Morphine milligram equivalents; ICU: Intensive care unit; TXA: Tranexamic acid; KSS: Knee Society Score; VAS: Visual analogue scale; WOMAC: Western Ontario and McMaster Universities Osteoarthritis Index; KOOS: Knee injury and Osteoarthritis Outcome Score; SF-36: 36-Item Short Form Health Survey

Baseline demographic and clinical characteristics are presented in Table 2. The mean age of patients ranged from 59.3 to 80.3 years, with a pooled average of 67.9 ± 4.1 years; patients in the simultaneous groups were generally slightly younger than those in the staged groups. Across all studies, male patients represented 41.5% of the total population, confirming that females were the majority in both surgical strategies. BMI ranged from 25 to 33 kg/m^2^, with no meaningful differences between groups.

**Table 2:** Baseline demographics and clinical characteristics of patients undergoing simultaneous versus staged bilateral TKA

Studies	Interventions	Mean age ± SD (years)	Number of male (%)	Mean Charlson comorbidity index	Mean BMI	Mean ASA score
Singh et al. [14]	Simultaneous *N* = 89,568	63.51 ± 8.38	42,336 (47%)	Elixhauser index: 0.16 ± 4.13	N/A	N/A
	Staged *N* = 121,115	65.51 ± 8.92	48,509 (40%)	Elixhauser index: 0.08 ± 4.27		
	*p* value	< 0.001	< 0.001	< 0.001		
Accatino et al. [15]	Simultaneous *N* = 43	70.2 [42–85]	14 (33%)	N/A	N/A	N/A
	Staged *N* = 66	64.2 [48–75	30 (45%)			
	*p* value	0.001219	0.2201			
Ashkenazi et al. [16]	Simultaneous *N* = 205	61.6 (26–80)	240 (40.3%)	2.1 ± 1.6	31.0 (18.6–86.9)	1: 25 (4.2%) 2: 527 (88.7%) 3: 42 (7.1%) 4: 0
	Staged *N* = 205	65.2 (21–92)	477 (27.1%)	2.8 ± 1.9	33.6 (16.3–67.5)	1: 27 (1.5%) 2: 1003 (57.2%) 3: 708 (40.4%) 4: 16 (0.9%)
	*p* value	< 0.001	< 0.001	< 0.001	< 0.001	< 0.001
Chang et al. [17]	Simultaneous *N* = 1558	71.8 ± 6.9	423 (18.0%)	0: 11 (0.5%) 1: 54 (2.3%) 2: 435 (18.6%) 3: 840 (35.8%) 4: 607 (25.9%) 5: 272 (11.6%) 6+: 125 (5.3%)	28.2 ± 4.2	N/A
	Staged *N* = 786	71.0 ± 7.3	298 (19.1%)	0: 6 (0.4%) 1: 35 (2.2%) 2: 277 (17.8%) 3: 580 (37.2%) 4: 411 (26.4%) 5: 170 (10.9%) 6+: 79 (5.1%)	28.3 ± 4.4	
	*p* value	0.010	0.055	0.140	0.889	
Franceschetti et al. [18]	Simultaneous *N* = 65	68 ± 9	23 (35%)	4 (3–6)	29.96 ± 5.20	2.25 ± 0.5
	Staged *N* = 108	70 ± 7	32 (30%)	4 (3–5)	30.79 ± 4.06	2.24 ± 0.4
	*p* value	0.039	0.501	0.754	0.196	0.870
Kim et al. [19]	Simultaneous *N* = 7155	67 (55–89)	2933 (69.4%)	1: 1431 (20%) 2: 3577 (50%) 3: 1073 (15%) 4: 1074 (15%)	28 (25–38)	1: 1503 (21%) 2: 3506 (49%) 3: 1145 (16%) 4: 1001 (14%)
	Staged > 1 week *N* = 4501	69 (58–92)	1890 (72.3%)	1: 855 (19%) 2: 2070 (46%) 3: 720 (16%) 4: 856 (19%)	30 (27–38)	1: 949 (21%) 2: 2160 (48%) 3: 540 (12%) 4: 852 (19%)
	*p* value	0.105	0.211	Not significant	0.171	Not significant
Matsumura et al. [20]	Simultaneous *N* = 94	76 (45–90)	77 (18%)	1–2: 52 (12%) 3–4: 267 (62%) 5+: 115 (26%)	26 (13–49)	1: 67 (15%) 2: 341 (79%) 3: 26 (6%)
	Staged *N* = 94	76 (44–91)	19 (20%)	1–2: 2 (2%) 3–4: 42 (43%) 5+: 53 (55%)	25 (14–38)	1: 12 (12%) 2: 83 (86%) 3: 2 (2%)
	*p* = value	0.694	0.672	< 0.001	0.043	0.166
Serino et al. [21]	Simultaneous *N* = 7454	63.5 ± 8.0	3363 (45.6%)	Elixhauser index: 4.44 ± 2.83	N/A	N/A
	Staged 90–365 days *N* = 44,196	66.3 ± 8.2	13,271 (34%)	Elixhauser index: 4.99 ± 3.01		
	*p* value	N/A	N/A	N/A		
Sustich et al. [22]	Simultaneous *N* = 23	63 (57–67)	16 (70%)	N/A	27.4 (26.1–33.1)	N/A
	Staged *N* = 50	70 (64–77)	33 (66%)		30.4 (27.4–34.7)	
	*p* value	< 0.001	1		0.165	
Tsui et al. [23]	Simultaneous *N* = 772	66.4 ± 7.50	205 (26.6%)	0.02	N/A	1: 2905 (90.8%) 2: 245 (7.66%) 3: 55 (91.56%)
	Staged *N* = 1600	70.4 ± 7.99	387 (24.2%)	0.08		1: 719 (93.1%) 2: 48 (6.22%) 3: 5 (0.65%)
	*p* value	< 0.001	Not significant	< 0.001		N/A
Yalin et al. [24]	Simultaneous *N* = 48	64.8 (51–81)	10 (20.8%)	3.6 (3–5)	27.4 (25–29)	1.8 (1–3)
	Staged *N* = 114	68.2 (53–88)	15 (13.1%)	3.7 (3–5)	27.97 (26–32)	2.05 (1–3)
	*p* value	N/A	N/A	N/A	N/A	N/A
Abdalla et al. [25]	Simultaneous *N* = 27	65.22 ± 8.77	7 (25.9%)	N/A	31.24 ± 1.6	N/A
	Staged *N* = 26	67.81 ± 6.86	9 (34.6%)		32 ± 1.25	
	*p* value	0.237	0.491		0.539	
Celen et al. [26]	Simultaneous *N* = 168	67.3 ± 6.5	26 (15.4%)	N/A	33.2 ± 5.2	1: 57 (34%) 2: 77 (45.8%) 3: 34 (20.2%)
	Staged *N* = 63	67.1 ± 6.9	12 (19%)		34.1 ± 4.5	1: 20 (31.7%) 2: 24 (38.1%) 3: 19 (30.2%)
	*p* value	0.793	0.514		0.228	0.265
Chou et al. [3]	Simultaneous *N* = 1565	72.2 ± 8.0	351 (22.4%)	N/A	28.0 ± 4.2	1: 13 (1.9%) 2422 (62.3%) 3: 235 (34.7%) 4: 7 (1.0%)
	Staged *N* = 451	71.9 ± 9.0 (63–97)	99 (22.0%)		28.6 ± 4.4	1: 7 (3.0%) 2: 135 (57.2%) 3: 91 (38.6%) 4: 3 (1.3%)
	*p* value	0.39	0.83		< 0.01	Not significant
Erossy et al. [27]	Simultaneous *N* = 19,382	65 ± 9	7990 (41.2%)	N/A	N/A	N/A
	Staged *N* = 19,382	65 ± 9	7937 (41%)			
	*p* value	0.83	0.5843			
Eke et al. [28]	Simultaneous *N* = 225	66.88 ± 5.79	34(15.1%)	N/A	N/A	N/A
	Staged *N* = 51	69.52 ± 6.7	12(24%)			
	*p* value	0.056	0.135			
Abdelaal et al. [29]	Simultaneous *N* = 2728	63.2 [57.5–69.5]	1099 (40.3%)	0.27 ± 0.62	30.5 [27.3–34.5]	N/A
	Staged *N* = 829	67.8 [61.3–74.6]	540 (37.0%)	0.55 ± 0.94	32.3 [28.1–37.8]	
	*p* value	< 0.001	0.039	< 0.001	< 0.001	
Mardani-Kivi et al. [30]	Simultaneous *N* = 272	40–50: 83 (30.51%) 50–60: 113 (41.54%) 60–75: 76 (27.94%)	78 (28.67%)	N/A	20–25: 27(9.93%) 25–30: 78 (28.68%) 30–35: 137 (50.36%) 35+: 30 (11.02%)	N/A
	Staged 1–12 months *N* = 245	40–50: 78 (31.83%) 50–60: 96 (39.18%) 60–75: 71 (28.97%)	83 (33.87%)		20–25: 25 (10.2%) 25–30: 69 (28.16%) 30–35: 118 (48.16%) 35+: 33 (13.47%)	
	*p* value	0.929	0.759		0.999	
Sarazeem et al. [31]	Simultaneous *N* = 51	62.4 ± 4.51	5 (9%)	N/A	25.8 ± 2.48	N/A
	Staged *N* = 49	61.70 ± 5.62	5 (10%)		26.1 ± 7.59	
	*p* value	0.503	0.001		0.879	
Gill et al. [32]	Simultaneous *N* = 122	70.6 ± 7.1	46 (37.7%)	1 (0–2)	N/A	1: 7 (5.8%) 2: 81 (66.9%) 3: 33 (27.3%)
	Staged *N* = 46	70.7 ± 8.8	23 (50%)	1 (0–2)		1: 1 (2.2%) 2: 31 (67.4%) 3: 14 (30.4%)
	*p* value	0.904	0.149	0.345		0.682
Richardson et al. [33]	Simultaneous *N* = 1637	< 49: 1.34% 50–59: 11.12% 60–69: 41.42% 70–79: 38.36% 80–89: 6.6% > 90: 1.16%	44.17%	N/A	N/A	N/A
	Staged 6–12 months *N* = 2695	< 49: 1.19% 50–59: 9.87% 60–69: 37.7% 70–79: 41.26% 80–89: 8.68% > 90: 1.3%	34.03%			
	*p* value	0.009	< 0.001			
Tsay et al. [34]	Simultaneous *N* = 27,301	< 50: 1095 (4%) 60–64:10,734 (39.3%) 65–74: 10,344 (37.9%) > 74: 11,807 (43.2%)	11,807 (43.2%)	0: 591 (2.2%) 1–2:11,234 (41.1%) 3–4: 12,056 (44.2%) 5+: 3420 (12.5%)	N/A	N/A
	Staged *N* = 45,419	< 50: 1938 (4.3%) 60–64: 16,211 (35.7%) 65–74: 17,372 (38.2%) > 74: 9898 (21.8%)	16,980 (37.4%)	0: 619(1.4%) 1–2: 138,875 (30.6%) 3–4: 21,462 (47.3%) 5+: 9463 (20.8%)		
	*p* value	N/A	N/A	N/A		
Chua et al. [35]	Simultaneous *N* = 23,136	< 55: 2284 (9.9%) 55–64: 8526 (36.9%) 65–74: 8979 (38.8%) > 75: 3347 (14.5%)	12,449 (53.8%)	N/A	Underweight: 10.9% Normal:10% Pre-obese: 29.5% Obese class 1: 29.4% Obese class 2: 13.7% Obese class 3: 6.5%	N/A
	Staged 3–6 months *N* = 9051	< 55: 544 (6.0%) 55–64: 2554 (28.2%) 65–74: 3628 (40.1%) > 75: 2325 (25.7%)	4388 (48.5%)		Underweight: 12.9% Normal: 8.4% Pre-obese: 26.2% Obese class 1: 19.8% Obese class 2:19% Obese class 3: 25.8%	
	*p* value	N/A	N/A		N/A	
Koh et al. [36]	Simultaneous *N* = 820	68.6 ± 5.9	34 (4.1%)	N/A	27.0 ± 3.4	1: 19 (5.2%) 2: 325 (88.3%) 3: 24 (6.5%)
	Staged > 1 week *N* = 265	69.9 ± 7.1	13 (4.9%)		27.1 ± 3.7	1: 46 (5.6%) 2: 763 (93.1%) 3: 11 (1.3%)
	*p* value	0.004	0.692		0.929	< 0.001
Bohm et al. [37]	Simultaneous *N* = 6349	64 (58–71)	2603 (41%)	N/A	N/A	N/A
	Staged *N* = 25,253	66 (60–73)	9849 (39%)			
	*p* value	< 0.001	0.01			
Sheth et al. [38]	Simultaneous *N* = 7866	64.94 ± 8.86	42.72%	N/A	31.58 ± 6.11	2.32 ± 0.52
	Staged *N* = 14,370	66.84 ± 8.93	36.6%		32.70 ± 6.54	2.4 ± 0.53
	*p* value	N/A	N/A		N/A	N/A
Courtney et al. [39]	Simultaneous *N* = 103	59.4 ± 9.6	38 (37%)	0.53 ± 0.9	31.7 ± 6.6	2.34 ± 0.55
	Staged *N* = 131	64.2 ± 9.7	30 (23%)	1.06 ± 1.2	36 ± 9.2	2.51 ± 0.55
	*p* value	< 0.001	0.064	< 0.001	< 0.001	0.027
Lindberg-Larsen et al. [40]	Simultaneous *N* = 157	64.0 (8.2)	47.1%	N/A	N/A	N/A
	Staged 7–18 months *N* = 292	66.7 (9.2)	34.2%			
	*p* value	N/A	N/A			
Niki et al. [41]	Simultaneous *N* = 60	73.0 ± 7.9	10 (16.6%)	N/A	26.4 ± 3.5	N/A
	Staged *N* = 60	72.3 ± 7.1	9 (15%)		25.5 ± 4.3	
	*p* value	Not significant	Not significant		Not significant	
Bolognesi et al. [42]	Simultaneous *N* = 4519	73.3 ± 5.3	1853 (41.0%)	N/A	N/A	N/A
	Staged *N* = 3788	74.1 ± 5.7	1466 (38.7%)			
	*p* value	< 0.001	0.033			
Poultsides et al. [43]	Simultaneous *N* = 2825	65.2 ± 9.0	1062 (37.6%)	0.35 ± 0.812	N/A	N/A
	Staged *N* = 1151	69.5 ± 9.2	371 (37.6%)	0.66 ± 1.132		
	*p* value	< 0.001	0.006	< 0.001		
Bini et al. [44]	Simultaneous *N* = 1220	< 64: 1076 (45.1%) > 65: 1603 (38.5%)	996 (41.6%)	N/A	< 29: 613 (46.1%) 30–34: 376 (28.3%) > 35: 341 (25.6%)	1–2: 1524 (66.2%) 3: 778 (33.8%)
	Staged *N* = 2116	< 64: 1311 (54.9%) > 65: 2563 (61.5%)	1416 (34.2%)		< 29: 919 (38.2%) 30–34: 699 (29.1%) > 35: 785 (32.7%)	1–2: 2517 (62.9%) 3: 1483 (37.1%)
	*p* value	< 0.001	< 0.001		< 0.001	0.009
Yoon et al. [45]	Simultaneous *N* = 119	70 (34–83)	7 (5.9%)	N/A	26.4 (19.1–34.2)	N/A
	Staged *N* = 119	70 (34–83)	7 (5.9%)		26.5 (18.3–35.6)	
	*p* value	N/A	N/A		N/A	
Stefansdottir et al. [46]	Simultaneous *N* = 1139	74.3 (67.4–85.0)	40.8%	N/A	N/A	N/A
	Stage > 1 year stage 2 *N* = 6560	80.3 (70.5–89.0)	N/A			
	*p* value	N/A	N/A			
Forster et al. [47]	Simultaneous *N* = 28	66 (51–70)	15 (53.5%)			1.8 (1–3)
	Staged 1 week apart *N* = 36	68 (48–77)	18 (50%)			2 (1–3)
	Staged > 1 week apart *N* = 38	64 (41–79)	16 (42.1%)			2 (1–3)
	*p* value	N/A	N/A			N/A
Hutchinson et al. [48]	Simultaneous *N* = 438	67	56%	N/A	N/A	N/A
	Staged *N* = 125	65	35%			
	*p* value	0.288	< 0.001			
Walmsley et al. [49]	Simultaneous *N* = 826	N/A	N/A	N/A	N/A	N/A
	Staged *N* = 1796					
	*p* value					
Silva et al. [50]	Simultaneous *N* = 26	59.3 (41–76)	14 (54%)	N/A	N/A	N/A
	Staged > 7 days *N* = 65	67.2 (48–90)	14 (22%)			
	*p* value	N/A	N/A			
Stubbs et al. [51]	Simultaneous *N* = 61	66	N/A	N/A	N/A	N/A
	Staged *N* = 76	65				
	*p* value	N/A				
Ritter et al. [52]	Simultaneous *N* = 2050	69.9	906 (44%)	N/A	N/A	N/A
	Staged *N* = 152	69.2	35 (23%)			
	*p* value	N/A	N/A			
Mangaleshkar et al. [53]	Simultaneous *N* = 54	< 75: 53.7% > 75: 46.3%	38.9%	N/A	N/A	1: 6 (11.1%) 2: 31 (57.4%) 3: 17 (31.5%)
	Staged *N* = 34	< 75: 70.59% > 75: 29.41%	38.2%			1: 3 (8.8%) 2: 21 (61.8%) 3: 10 (29.4%)
	*p* value	N/A	N/A			N/A
Ritter et al. [54]	Simultaneous *N* = 12,922	73.4	38.6%	N/A	N/A	N/A
	Staged 6–12 months *n* = 31,401	72.7	30.3%			
	*p* value	N/A	N/A			

Comorbidity burdens, assessed using Charlson or Elixhauser indices, were overall modest and comparable between strategies. The pooled Charlson Index averaged 1.83 ± 1.48 (range 0.27–4.0), while the Elixhauser Index averaged 2.42 ± 2.66 (range 0.08–4.99). Although staged cohorts occasionally showed slightly higher values, differences were small, inconsistently significant across datasets, and some studies did not report these measures. ASA scores were predominantly grades 2–3 in both groups.

Outcomes assessed included perioperative parameters (operative time, blood loss, transfusions), complications (neurologic, cardiovascular, pulmonary, genitourinary, thrombotic, infectious, arthrofibrosis, extensor-mechanism failure, periprosthetic fracture), functional scores (Knee Society, Oxford, WOMAC, Forgotten Joint, HSS), readmissions, reoperations, and mortality (in-hospital, first- and second-year).

### Methodological quality assessment

**Fig. 2:**
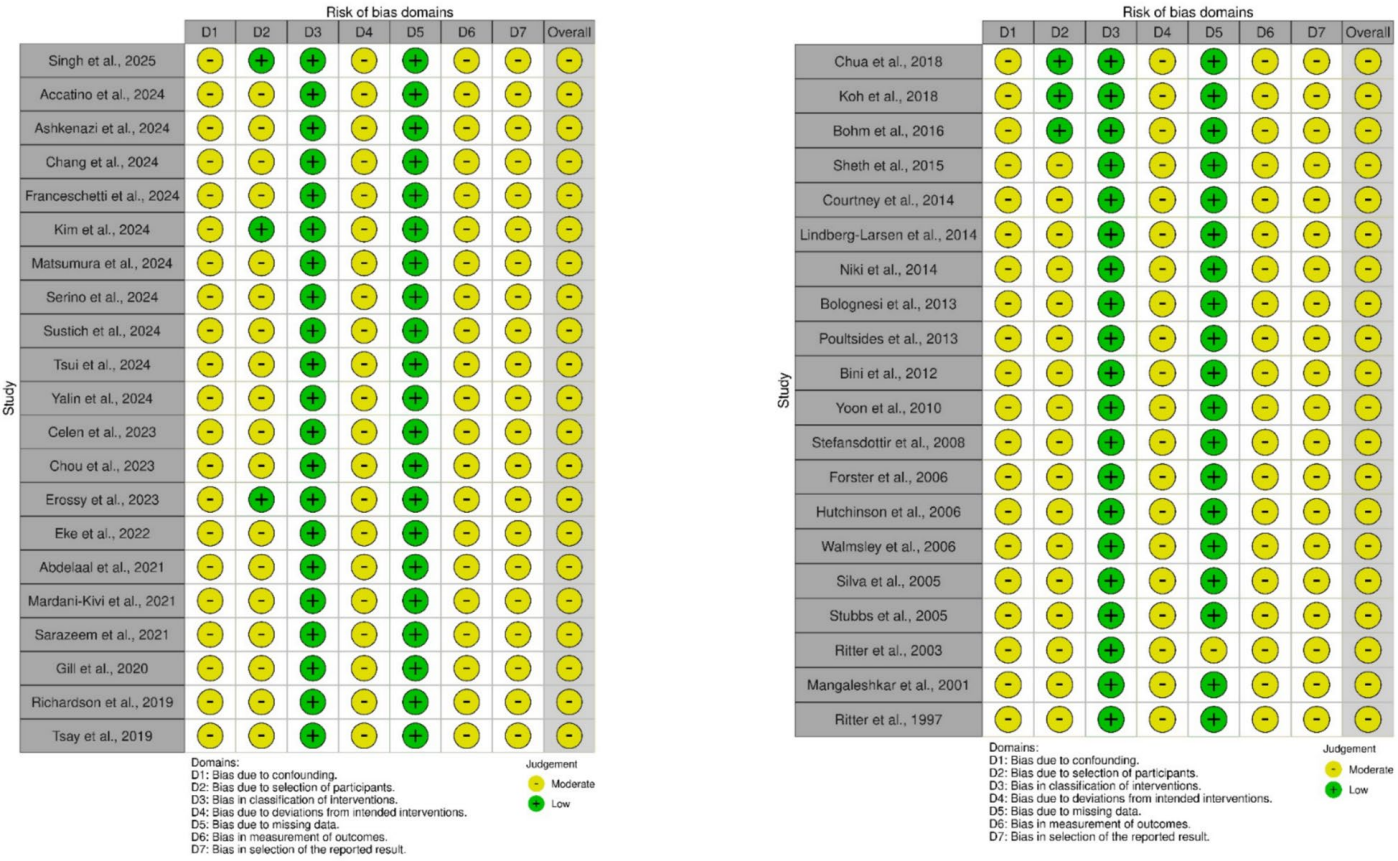
Risk of bias assessment of studies using the Cochrane Risk of Bias in Non-randomized Studies of Interventions (ROBINS-I) tool

**Fig. 3:**
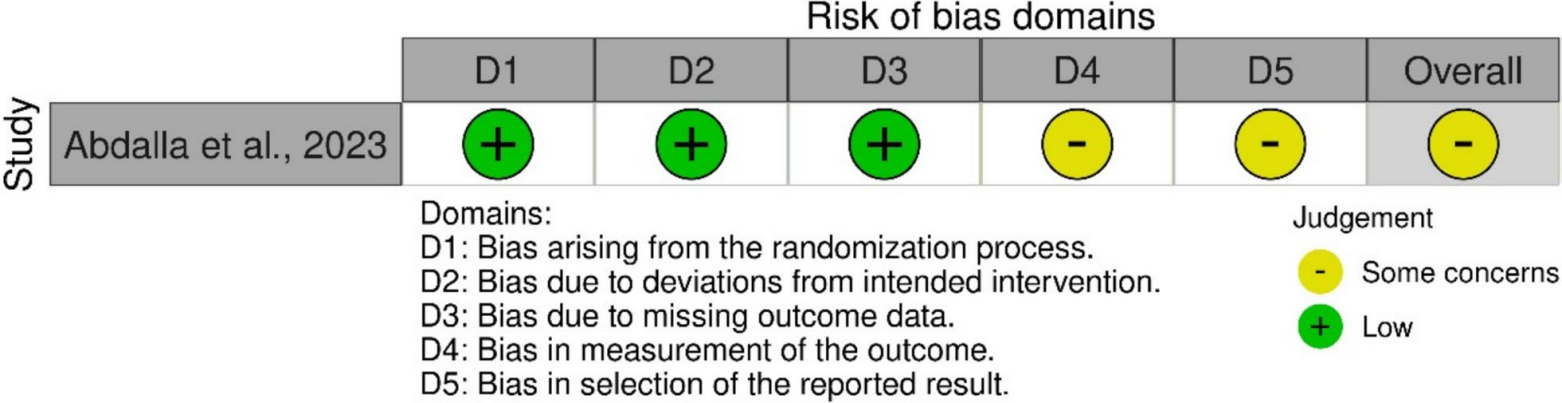
Risk of bias assessment of studies using the Cochrane Risk-of-Bias tool (RoB 2) tool

For the 41 non‐RCT cohorts (Fig. 2), ROBINS-I indicated a generally moderate overall risk of bias—most often driven by confounding—while domains such as intervention classification, outcome measurement, and reporting were largely judged at low or moderate concern. The single randomized trial (Fig. 3), assessed with ROB 2, demonstrated low risk in randomization and missing data but retained some concerns around deviations from intended interventions and selective outcome reporting.

### In-hospital complications

**Fig. 4:**
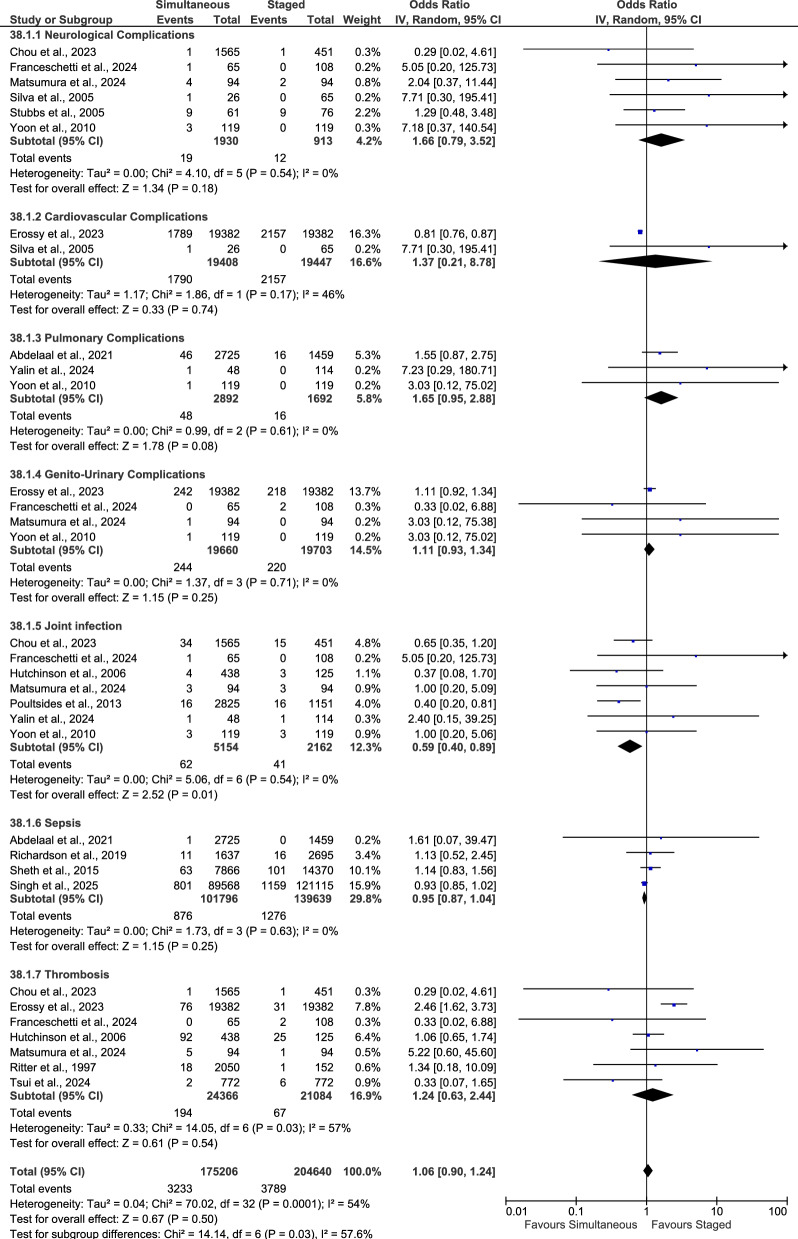
Forest plot of in-hospital complication rates

Using a random-effects model (Fig. 4), simultaneous and staged bilateral TKA were compared across seven in-hospital complication categories: neurological (cerebrovascular event, hemiparesis/paralysis, nerve disorders, delirium, stroke), cardiovascular (myocardial ischemia, cardiac arrest, heart failure, atrial fibrillation, arrhythmia, arterial dissection, angina), pulmonary (pneumonia, respiratory depression, respiratory failure), genito-urinary (renal failure, urinary retention, urinary tract infection, acute kidney injury), joint infection, sepsis, and thrombotic events (deep vein thrombosis, venous thromboembolism). There was no statistically significant difference between groups in in-hospital neurological complications (*p* = 0.18), cardiovascular complications (*p* = 0.74), pulmonary complications (*p* = 0.08), genito-urinary complications (*p* = 0.25), sepsis (*p* = 0.25), or thrombotic events (*p* = 0.54). Joint infection was significantly lower with simultaneous procedures (OR 0.59; 95% CI 0.40–0.89; *p* = 0.01).

### First-year complications

**Fig. 5:**
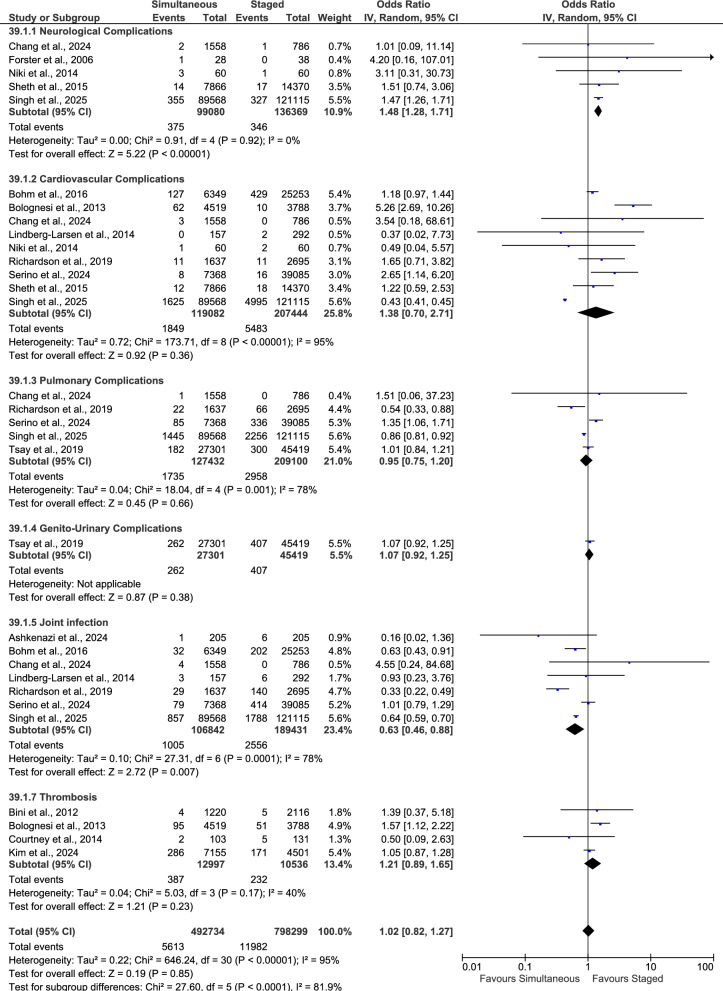
Forest plot of first-year complication rates

Using a random-effects model (Fig. 5), first-year post-discharge complications were compared between simultaneous and staged bilateral TKA across five complication categories. Neurological complications were significantly higher with simultaneous procedures (OR 1.48; 95% CI 1.28–1.71; *p* < 0.001). Cardiovascular (*p* = 0.36), pulmonary (*p* = 0.66), genito-urinary (*p* = 0.38), and thrombotic events (*p* = 0.23) showed no statistically significant differences between simultaneous and staged procedures.

### Complications between 1 and 2 years

**Fig. 6:**
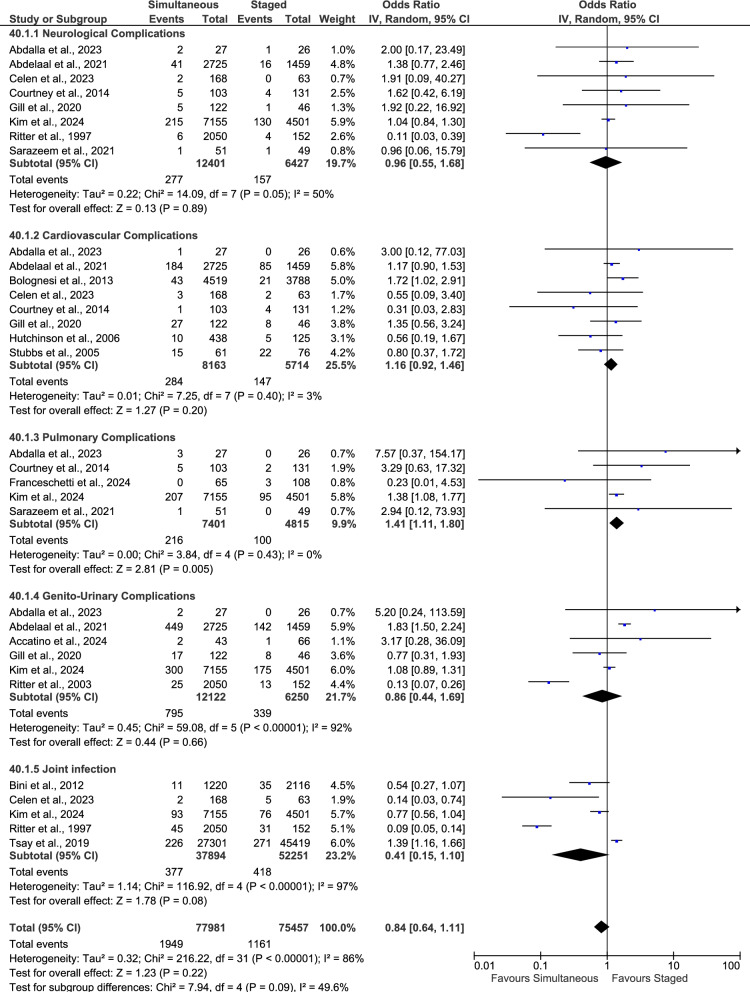
Forest plot of complication rates between 1 and 2 years

Using a random-effects model (Fig. 6), complications occurring between the first and second postoperative years were compared between simultaneous and staged bilateral TKA. Neurological (*p* = 0.89), cardiovascular (*p* = 0.20), and genito-urinary (*p* = 0.66) complications showed no statistically significant differences between groups, while pulmonary complications were higher with simultaneous procedures (OR 1.41; 95% CI 1.11–1.80; *p* = 0.005). Joint infection showed no statistically significant difference (*p* = 0.08). Overall, the pooled analysis demonstrated no significant difference in aggregate complication rates at 1–2 years (*p* = 0.22).

### In-hospital transfusion

**Fig. 7:**
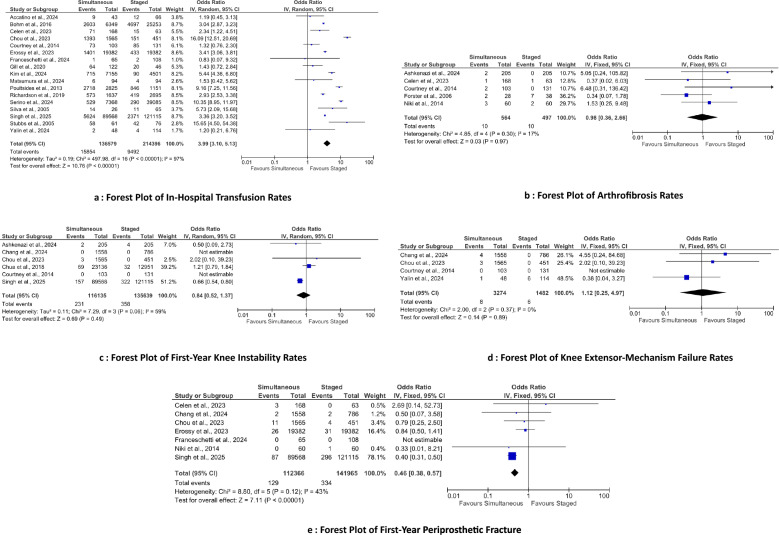
Comparison of perioperative complications between simultaneous and staged bilateral total knee arthroplasty (TKA). **a** Forest plot of in-hospital transfusion rates. **b** Forest plot of arthrofibrosis rates. **c** Forest plot of first-year knee instability rates. **d** Forest plot of knee extensor‐mechanism failure rates. **e** Forest plot of first-year periprosthetic fracture

A total of 17 studies including 350,975 patients (136,579 simultaneous, 214,396 staged) reported on in-hospital transfusion requirement (Fig. 7a). The pooled OR (random-effects model) was 3.99 (95% CI 3.10–5.13; *p* < 0.001), indicating that simultaneous bilateral TKA was associated with a significantly higher likelihood of transfusion compared with staged procedures.

### Arthrofibrosis

Five studies involving 1061 patients (564 simultaneous, 497 staged) reported arthrofibrosis between 1 and 18 months postoperatively (Fig. 7b). The pooled analysis (fixed‐effect model) showed no statistically significant difference in arthrofibrosis between simultaneous and staged bilateral TKA (*p* = 0.97).

### First-year knee instability

Four studies involving 251,774 patients (116,135 simultaneous, 135,639 staged) reported knee instability within the first year (Fig. 7c). The pooled analysis (random-effects model) showed no statistically significant difference between simultaneous and staged bilateral TKA (*p* = 0.49).

### Extensor‐mechanism failure

Four studies involving 4756 patients (3274 simultaneous, 1482 staged) reported extensor‐mechanism failure within the first year postdischarge (Fig. 7d). The pooled analysis (fixed‐effect model) showed no statistically significant difference (*p* = 0.89).

### First-year periprosthetic fracture

Seven studies including 254,331 patients (112,366 simultaneous, 141,965 staged) reported periprosthetic fractures occurring within the first postoperative year (Fig. 7e). The pooled OR (fixed‐effect model) was 0.46 (95% CI 0.38–0.57; *p* < 0.001), indicating that simultaneous bilateral TKA was associated with a significantly lower odds of periprosthetic fracture within 1 year compared with staged procedures.

### Mortality

**Fig. 8:**
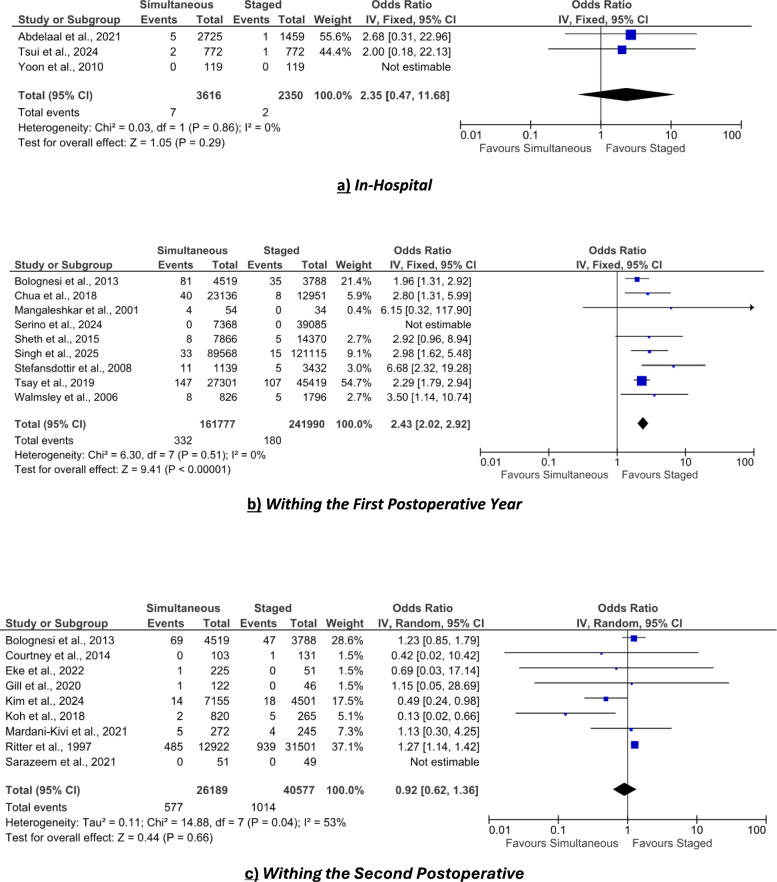
Forest plot of mortality rates: **a** in-hospital, **b** within the first postoperative year, and **c** within the second postoperative year

Three sets of studies assessing mortality were analyzed: in-hospital (three studies; 3616 simultaneous, 2350 staged), first-year (nine studies; 161,777 simultaneous, 241,990 staged), and second-year (nine studies; 26,189 simultaneous, 40,577 staged) (Fig. 8). Data were pooled using a fixed-effect model. In-hospital mortality showed no statistically significant difference (*p* = 0.29). First-year mortality was significantly higher with simultaneous procedures (OR 2.43; 95% CI 2.02–2.92; *p* < 0.001). Second-year mortality showed no statistically significant difference (*p* = 0.66).

### Persistent pain

**Fig. 9:**
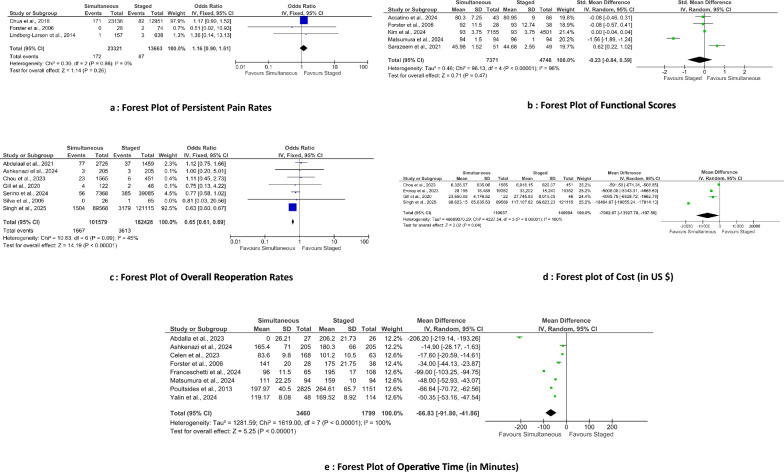
Forest plot comparing functional outcomes, reoperation rates, and healthcare resource utilization between simultaneous versus staged bilateral TKA. **a** Forest plot of persistent pain rates. **b** Forest plot of functional scores. **c** Forest plot of overall reoperation rates. **d** Forest plot of cost (in US$). **e** Forest plot of operative time (in min)

Three studies involving 36,984 patients (23,321 simultaneous, 13,663 staged) reported persistent pain at 30 days postsurgery (Fig. 9a). The pooled analysis (fixed‐effect model) showed no statistically significant difference (*p* = 0.26).

### Functional scores

Five studies involving 12,119 patients (7371 simultaneous, 4748 staged) reported function using the Knee Society Score or Oxford Knee Score (Fig. 9b). The pooled analysis (random-effects model) indicated no statistically significant difference (*p* = 0.47) in function between simultaneous and staged procedures.

### Overall reoperation

Six studies involving 264,005 patients (101,579 simultaneous, 162,426 staged) reported on overall reoperation rates. The pooled OR (fixed‐effect model) was 0.65 (95% CI 0.61–0.69; *p* < 0.001), indicating that simultaneous bilateral TKA was associated with a significantly lower odds of reoperation compared with staged procedures (Fig. 9c).

### Cost

Four studies involving 251,631 patients (110,637 simultaneous, 140,994 staged) reported in-hospital cost in US dollars. Costs were pooled using a random-effects model, with a pooled MD of −$7062.67 (95% CI −$13,927.78 to −$197.56; *p* = 0.04), indicating that simultaneous bilateral TKA was associated with significantly lower cost compared with staged procedures (Fig. 9d).

### Operative time

Eight studies involving 5295 patients (3460 simultaneous, 1799 staged) reported operative time. Data were pooled using a random‐effects model, yielding a pooled MD of −66.83 min (95% CI −91.80 to −41.86; *p* < 0.001), indicating that simultaneous bilateral TKA was associated with a significantly shorter operative time compared with staged procedures (Fig. 9e).

## Discussion

Our meta-analysis demonstrates that while overall perioperative outcomes of simultaneous and staged bilateral total knee arthroplasty are broadly comparable, several critical insights emerge that add new depth to the debate. On one hand, simultaneous bilateral TKA was associated with reduced reoperation rates and hospital costs, as well as lower risks of infection and periprosthetic fracture compared with staged procedures. On the other hand, these benefits were counterbalanced by higher transfusion requirements, increased first-year neurological complications, more pulmonary complications at 1–2 years, and a modest but measurable increase in early mortality. Together, these contrasting findings highlight the balance of risks and benefits between the two strategies. Importantly, our study incorporates 42 studies and over half a million patients, whereas all previous meta-analyses included far fewer articles and markedly smaller patient numbers [6, 55–60]. In addition, these earlier reviews did not capture many of the outcomes we report here, making our analysis both broader and more contemporary. This expanded evidence base strengthens the precision of our findings and underscores the clinical relevance of re-evaluating one-stage versus two-stage bilateral TKA. Taken together, these trade-offs explain why no single approach demonstrates clear superiority in the literature; rather, the “optimal” strategy is highly patient-dependent.

Notably, one-stage bilateral TKA was associated with more than twice the chance of requiring allogeneic blood transfusion during the index hospitalization. This reflects the greater cumulative blood loss of two knees being operated in one session, a finding consistently reported in prior series and large cohort analyses [14, 19, 61, 62]. It is worth noting that tranexamic acid, an antifibrinolytic agent commonly used to reduce surgical blood loss and transfusion requirements, was not consistently used or reported across the included studies. Only a minority of more recent studies adopted standardized intravenous protocols, which were generally applied equally in both simultaneous and staged groups. As such, the higher transfusion rates observed with simultaneous bilateral TKA likely reflect the inherent physiological burden of operating on both knees in a single setting. However, further studies with standardized and widely reported use of tranexamic acid are needed to clarify its impact on transfusion requirements in simultaneous versus staged procedures.

By contrast, the combined one-stage procedure did not significantly increase most in-hospital complication rates compared with staging the surgeries. Similarly, pooled data demonstrated that operative time was significantly shorter for simultaneous bilateral TKA, and that overall hospital costs were meaningfully reduced compared with staged procedures. Furthermore, our pooled analysis showed no statistically significant difference in neurological, pulmonary, or cardiovascular in-hospital complication rates between simultaneous and staged TKAs. Similarly, no significant difference in cardiovascular event rates was observed. These results suggest that, in contemporary practice, performing two knee replacements under one anesthetic can be accomplished without a marked increase in immediate postoperative morbidity for carefully selected patients.

Importantly, we found no evidence that one-stage bilateral TKA worsens postoperative joint outcomes or implant survival relative to staged surgery. Functional recovery at short-term follow-up was equivalent between the two strategies, indicating that patients achieve similar early improvements in pain relief and mobility whether both knees are replaced together or sequentially. Consistent with this, multiple studies have reported comparable postoperative clinical scores and patient-reported outcomes between simultaneous and staged bilateral knee arthroplasties [24, 63]. Even among older patients or those with higher perioperative risk profiles (ASA class 3), one-stage bilateral TKA can yield early outcomes and complication rates equivalent to staged protocols when managed in high-volume centers [63]. Furthermore, the aggregate “reoperation” rate was significantly lower with simultaneous bilateral surgery. In other words, patients who underwent a one-stage correction of both knees had a ~34% relative risk reduction in subsequent unplanned surgeries compared with those who had two separate operations. This finding aligns with recent large database analyses showing fewer return-to-operating-room events with one-stage bilateral procedures [14]. The reduced reoperation risk may stem from the avoidance of a second surgical episode and its attendant opportunities for complications (such as wound issues or prosthetic joint infection), as well as the elimination of interim “waiting period” failures of the first knee before the second is done. Notably, long-term overall reoperation rate appears to be comparable between one-stage and two-stage approaches. Taken together, these results indicate that a one-stage bilateral strategy does not compromise the durability of implants or functional recovery, while potentially even reducing the need for additional surgical interventions.

This study also sheds light on perioperative safety outcomes, particularly the risk of serious complications and mortality—outcomes that have historically driven the debate on simultaneous versus staged bilateral TKA. We noted a slight increase in early postoperative mortality with simultaneous bilateral TKA, although the absolute risk remained very low in both groups. Prior studies have similarly reported a higher 30-day mortality rate following simultaneous bilateral knee replacement [14, 56]. It should be noted that staged cohorts inherently exclude patients who suffer perioperative death or disabling complications after the first TKA and never proceed to the second—a form of survivorship bias that can make one-stage procedures seem riskier by comparison [35]. Nonetheless, the increased immediate physiological stress of two concurrent knee replacements remains a legitimate concern. Earlier large-cohort studies in the 2000s demonstrated higher rates of cardiac complications, pulmonary embolism, and stroke in patients undergoing simultaneous bilateral TKA, especially among older or medically complex individuals [6, 34]. We found no significant difference in aggregate thromboembolic events (deep vein thrombosis or pulmonary embolus) between simultaneous and staged groups in our pooled data, in contrast to some prior analyses that noted increased VTE with concurrent bilateral surgery [34, 58].

Furthermore, one-stage bilateral TKA carries certain distinct advantages. Patients in the simultaneous group avoided the need for a second hospitalization and anesthesia exposure, which may translate into significantly shorter total length of hospital stay and lower cumulative costs compared with two separate admissions [3, 24]. Chou et al. reported that staged bilateral TKA resulted in an average of five additional inpatient days and substantially higher overall medical expenditures relative to simultaneous surgery [3]. One-stage patients typically undergo a single rehabilitation period and return to baseline function sooner, whereas staged patients had to recover from two operations sequentially. Furthermore, performing both knees together minimizes duplicate preoperative workups and imaging, and has been shown to be cost-effective from the health system perspective [3, 24]. Some authors have noted that patient satisfaction is often higher when both arthritic knees are addressed in one setting, sparing the individual a protracted treatment course [18, 24]. However, these benefits must be weighed against the potential for increased short-term discomfort and dependency during the early recovery (since the patient cannot rely on an unoperated leg) and the aforementioned risk considerations.

Notably, our analysis and several recent studies found no increase in infection rates with simultaneous bilateral TKA [34, 58]. In fact, the pooled evidence suggests that one-stage surgery may reduce the risk of periprosthetic joint infection by limiting the patient’s cumulative exposure to hospital pathogens [14].

## Limitations

This meta-analysis has several limitations. First, most included studies were retrospective, so selection bias is likely. In addition, in staged cohorts a survivorship bias exists—patients must recover from the first TKA to receive the second, removing some high-risk cases from the staged group’s outcomes. Second, there was substantial heterogeneity across studies in patient populations, surgical techniques, perioperative protocols, TXA use, and outcome definitions. The interval between staged procedures varied widely, complicating direct comparisons. We used random-effects models to account for inter-study variability, but differences in practice patterns and temporal improvements in care limit the generalizability of our pooled estimates. Third, our meta-analysis relied on aggregate published data rather than individual patient-level data, limiting the ability to perform granular subgroup analyses (e.g. stratification by specific comorbidities or age). Outcomes reporting was inconsistent across studies, and publication bias is possible if studies with null findings were underreported. Finally, although the overall sample size was large, some rare but serious complications had low event rates, meaning our analysis may have been underpowered to detect very small between-group differences in those outcomes. Prospective randomized trials would best address selection bias, but such studies are logistically difficult; thus our conclusions derive from the best available observational evidence.

## Conclusions

This meta-analysis synthesizes the evidence that one-stage bilateral knee arthroplasty offers the benefit of a one-time rehabilitation, lower infection and periprosthetic fracture rates, fewer reoperations, and modest cost savings, at the price of higher transfusion requirements, increased neurological complications during the first postoperative year, more pulmonary complications between 1 and 2 years, and a modest but measurable rise in early mortality. Staged bilateral TKA, while spreading risk over two operations, incurs cumulative exposure to complications like infection, and not all patients complete the second stage. Therefore, surgeons and patients should engage in informed, individualized decision-making, armed with the quantitative evidence of this and other studies. By balancing the patient’s health status, priorities (one recovery versus two), and the outlined risks and benefits, the surgical team can choose the approach that maximizes benefit while minimizing harm. Ongoing research and improved risk stratification will hopefully continue to refine these recommendations, moving us closer to consensus on which patients are best served by simultaneous bilateral TKA and which should undergo staged procedures.

## Data Availability

Not applicable.
